# Field Testing of a Novel Drilling Technique to Expand Well Diameters at Depth in Unconsolidated Formations

**DOI:** 10.1111/gwat.13203

**Published:** 2022-05-06

**Authors:** Martin L. van der Schans, Martin Bloemendal, Niels Robat, Ate Oosterhof, Pieter J. Stuyfzand, Niels Hartog

**Affiliations:** ^1^ Faculty of Geoscience & Engineering Delft University of Technology Delft The Netherlands; ^2^ KWR Water Research Institute Nieuwegein The Netherlands; ^3^ Haitjema B.V Dedemsvaart The Netherlands; ^4^ Vitens N.V Zwolle The Netherlands; ^5^ Hydroconsult+ Zandvoort The Netherlands; ^6^ Department of Earth Sciences Utrecht University Utrecht The Netherlands

## Abstract

Larger well diameters allow higher groundwater abstraction rates. But particularly for the construction of wells at greater depth, it may be more cost‐efficient to only expand the borehole in the target aquifer. However, current drilling techniques for unconsolidated formations are limited by their expansion factors (<2) and diameters (<1000 mm). Therefore, we developed a new technique aiming to expand borehole diameters at depth in a controlled manner using a low‐pressure water jet perpendicular to the drilling direction and extendable by means of a pivoting arm. During a first field test, the borehole diameter was expanded 2.6‐fold from 600 to 1570 mm at a depth of 53.5 to 68 m and equipped with a well screen to create an expanded diameter gravel well (EDGW). In keeping with the larger diameter, the volume flux per m screen length was two times higher than conventional 860 mm diameter wells at the site in the subsequent 3 year production period. Although borehole clogging was slower on a volumetric basis and similar when normalized to borehole wall area, rehabilitation of particle clogging at the borehole wall was more challenging due to the thickness of the gravel pack. While jetting the entire borehole wall before backfilling holds promise to remove filter cake and thus limit particle clogging, we found that a second borehole (expanded 4.1‐fold to 2460 mm) collapsed during jetting. Overall, the EDGW technique has potential to make the use of deeper unconsolidated aquifers economically (more) feasible, although further understanding of the borehole stability and rehabilitation is required to assess its wider applicability.

## Abstract 2

*Article impact statement*: A novel drilling technique was developed and tested to expand the diameter of wells at depth during construction in unconsolidated aquifers.

## Introduction

Millions of drilled wells are used worldwide to abstract and inject groundwater (Margat and Van der Gun 2013; Fleuchaus et al. 2018; Dillon et al. 2019; Mukherjee et al. 2020; Jasechko and Perrone 2021). Due to the large capital expenditures required for well construction (Glotfelty 2017), maximum volume fluxes are an important design feature (Houben 2015a). These

volume fluxes are in practice often restricted by constraints to the entrance velocity, such as the design rules proposed by Sichardt (1928) and Huisman (1972) that limit the entrainment of fine grains from the formation, reduce clogging risks and curb turbulent losses. Despite the limited influence that well diameter has on drawdown, increasing the diameter is therefore often the only way to increase the volume flux of a well. Hence, with the high suitability of unconsolidated formations for wells (Domenico and Schwartz 1998; Pyne 2005; Bloemendal et al. 2015), different techniques have been developed for drilling large boreholes in such formations. These include forward reamers (Driscoll 1986), dual reverse circulation drilling (Anonymous 2006; Montiea 2015), augers (Johnson et al. 2009), and bucket excavators (Anonymous 2008). They allow for diameters that are substantially larger (up to 4000 mm) than the typically used 150 to 800 mm (Misstear et al. 2017; table 5.1).

**Figure 1:**
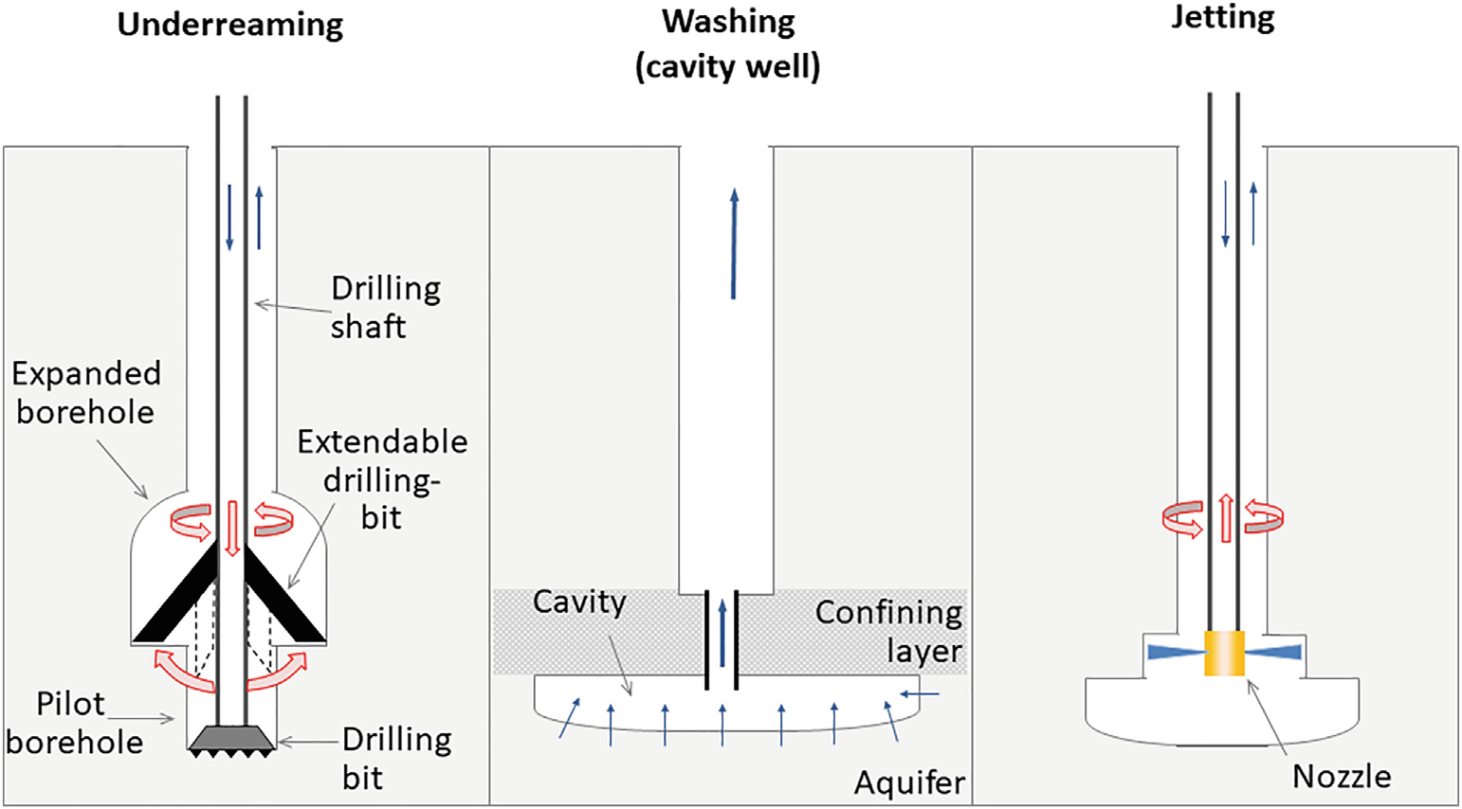
Illustration of existing mechanisms to enlarge drilled vertical boreholes for water wells at depth in unconsolidated formations. After: Driscoll (1986), Brown and Gledhill (2003), Fontenot et al. (2005) (underreaming), Saharawat et al. (2009) (washing), and Gao et al. (2015) (jetting).

Since drilling larger diameters leads to higher construction costs (Pan et al. 2020), being able to expand the borehole diameter only at the depth range targeted for the well screen can be an attractive option, especially at greater depths. Therefore, several expansion mechanisms have been developed (see Figure 1). However, these techniques either have a limited diameter expansion factor (<2) and limited diameter range (<914 mm) (underreamers: Mills Machine 2020, CaseyJones 2021), lack control over the diameter (jetting: Gao et al. 2015), or do not allow backfilling (cavity wells: Saharawat et al. 2009). Therefore, we aimed to develop a technique that allowed for borehole expansion ratios larger than factor 2 while maintaining control over the diameter and allowing for backfilling. We tested the developed expanded diameter gravel well (EDGW) technique in an unconsolidated fine sand aquifer.

## Background

As an overview of borehole expansion techniques relevant for construction of vertical water wells in unconsolidated formations is missing in scientific literature, we here provide their main characteristics.

### Underreaming

Underreaming (Figure 1) is a technique to expand boreholes that dates back at least to 1890 when a patent was issued involving the retrieving of a hydraulically expandable bit (Tessari and Madell 1999). It involves extension of cutting members against the wall of a pilot (=initial) borehole once the tool is positioned at the desired depth. The surrounding formation is then removed by pushing the expanded drilling bit down while rotating it (Driscoll 1986; Brown and Gledhill 2003). The borehole expansion ratio is limited to a factor 2 for sand and gravel formations with a maximum expanded diameter of 914 mm (Mills Machine 2020; CaseyJones 2021). Limitations are due to risks of mechanical failure inhibiting retraction of the blades and causing downhole loss of the drilling assemblies (Bruce 2012; Kamp 2018). Therefore, for consolidated formations, the maximum reported expansion ratio (1.3) (e.g., Brown and Gledhill 2003; Kerunwa and Anyadiegwu 2015; Schlumberger 2020) and diameter (406 mm) (Pavković et al. 2016) are lower. “Scrape drilling” is an underreaming technique with small expansion ratios (factor 1.05) intended to prevent initial clogging by scraping the filter cake of the borehole wall before backfilling (Olsthoorn and Harlingen 1994; Kortleve 1998; Segalen et al. 2005).

### Washing

Another mechanism to expand boreholes is washing (or eroding) the formation material by inducing a high water velocity at the borehole wall. Cavity wells involve washing out sand deposits underneath a clay layer by pumping water and sand through a cased pilot hole at high rate until a hollow cavity is formed. Cavity‐depths are reached up to 0.5 m and diameters up to 8000 mm (expansion ratio 32 for a 250 mm diameter pilot borehole). The large expansion ratio compared with cavity‐depths inhibits gravel packing, thus limiting lifespan to several years or decades due to collapse of the clay roof in the unsupported borehole (Thomas 1982; Taneja and Khepar 1996; Kamra et al. 2005).

Washouts (erosion) also occur unintendingly due to high flow velocities in the annular space or pressure jets in the drilling bit. They may cause uneven borehole enlargement, especially across strata with different solidity, thus undermining borehole stability (Chemerinski and Robinson 1995; Conn 2011; Maliva 2016).

### Jetting

Borehole expansion by jetting involves eroding formation material with a fluid that exits a nozzle at high velocity and pressure perpendicular to the drilling shaft (Lin et al. 2012; Shen et al. 2012). It is commonly applied to create grout foundation pillars in geotechnical applications (Bruce 1989). Gao et al. (2015) used high pressure (8–25 MPa) side nozzles in the center of the borehole to expand the diameter of lateral boreholes. They reached a diameter of 1.0 m in softer coal layers, compared with the normal range of 0.04 to 0.15 m (expansion ratio 6 to 25). However, the diameter was not only controlled by the applied pressure but also by the softness of the formation material, thus leading to an uneven distribution of the hole enlargement.

## Methods

### Conceptual Design: Extendable Jetting

**Figure 2:**
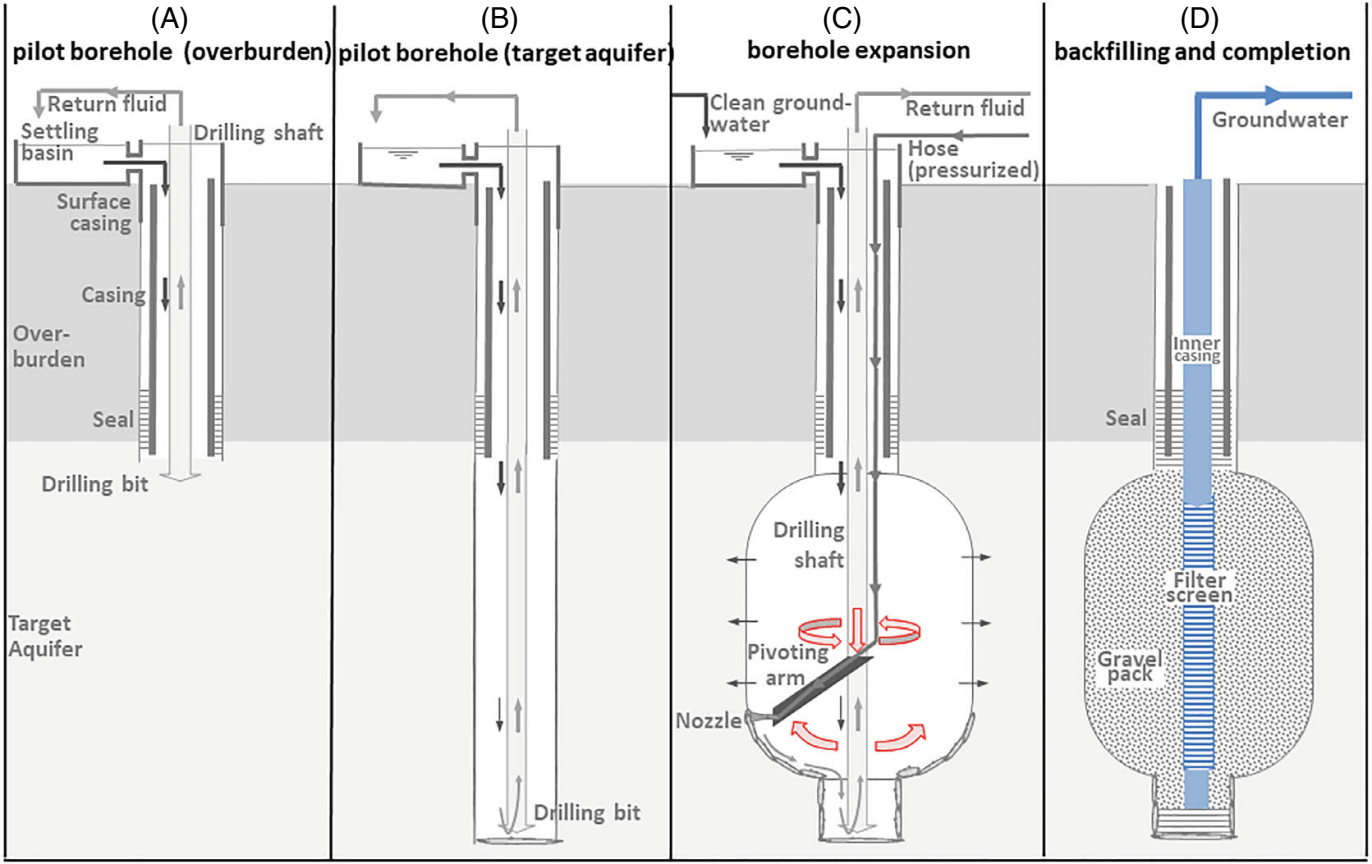
Illustration of the expanded jetting technique to borehole expansion using a pivoting arm equipped with a jetting nozzle (A–C) and its completion as an EDGW (D).

To allow expansion of a borehole at depth in a controlled manner, we designed a mechanism that involves low‐pressure jetting of a pilot borehole with a nozzle that is extended perpendicular to the drilling direction while rotating with the drilling shaft (Figure 2C). The waterjet is to expand the borehole by gently loosening and removing the formation material just in front of nozzle. The diameter of the borehole is thus controlled through the position of the nozzle relative to the center of the borehole, the volume flux and the diameter of the nozzle. The velocity flowing out of the nozzle needs to be sufficient to prevent mechanical drag by contact between the formation and the expansion arm. During expansion, the nozzle is continuously rotated and either moved up‐and‐down over the entire target depth or used to expand the borehole in vertical sections. The loosened formation materials (cuttings) are pumped to ground surface through the drilling shaft.

### Construction of the Extendable Jetting Nozzle

**Figure 3:**
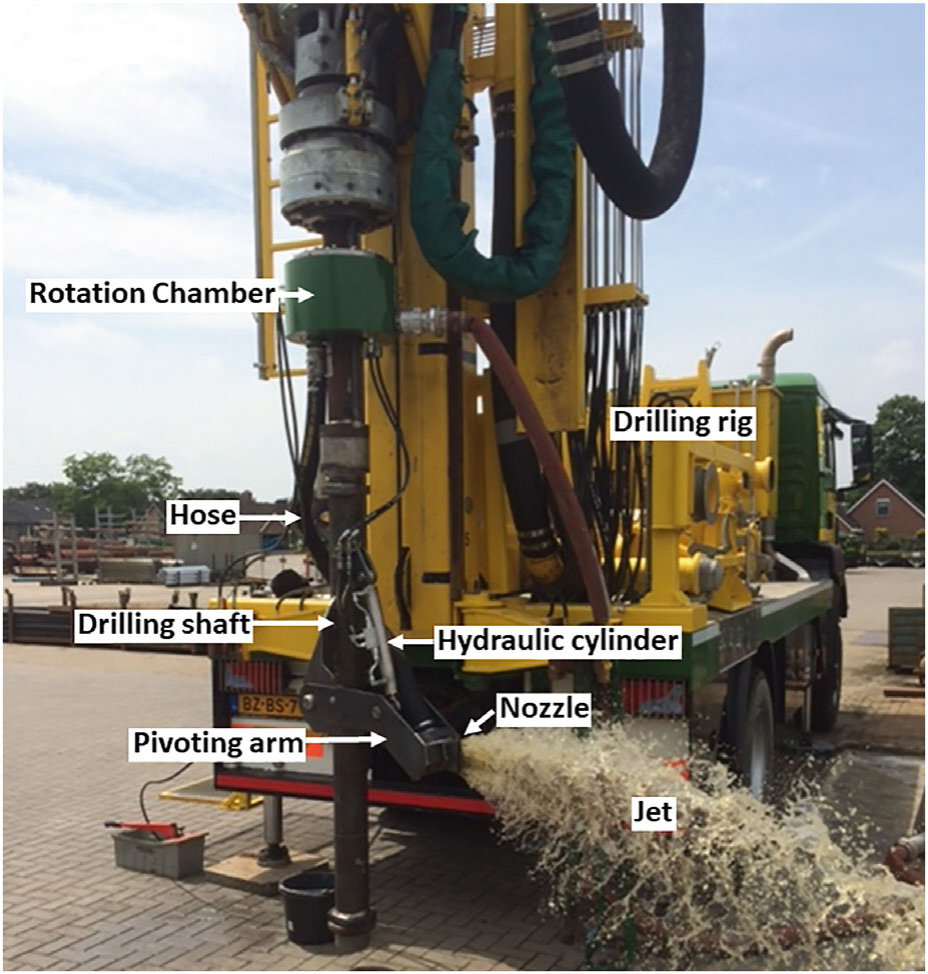
Illustration of the extension arm during aboveground testing.

A nozzle with a rectangular opening of 20 × 130 mm was attached to a steel arm that could be pivoted away from the drilling shaft by means of a hydraulic cylinder (Figure 3). The length of the arm (1.5 m) and maximum pivoting angle allowed a maximum 750 mm distance from the center of the borehole to the nozzle, with the aim of achieving a borehole diameter of just over 1500 mm. A rotation chamber was mounted at the top of the drilling shaft to allow rotation of the pivoting arm without entanglement of the water supply hose attached to the drilling shaft. Based on visual observation during an aboveground test in a water filled container, the nozzle discharge was limited to 11 m^3^/h at a pressure between 0.48 and 0.52 MPa at the hydraulic cylinder.

### Espelo Field Site and Hydrogeology

**Figure 4:**
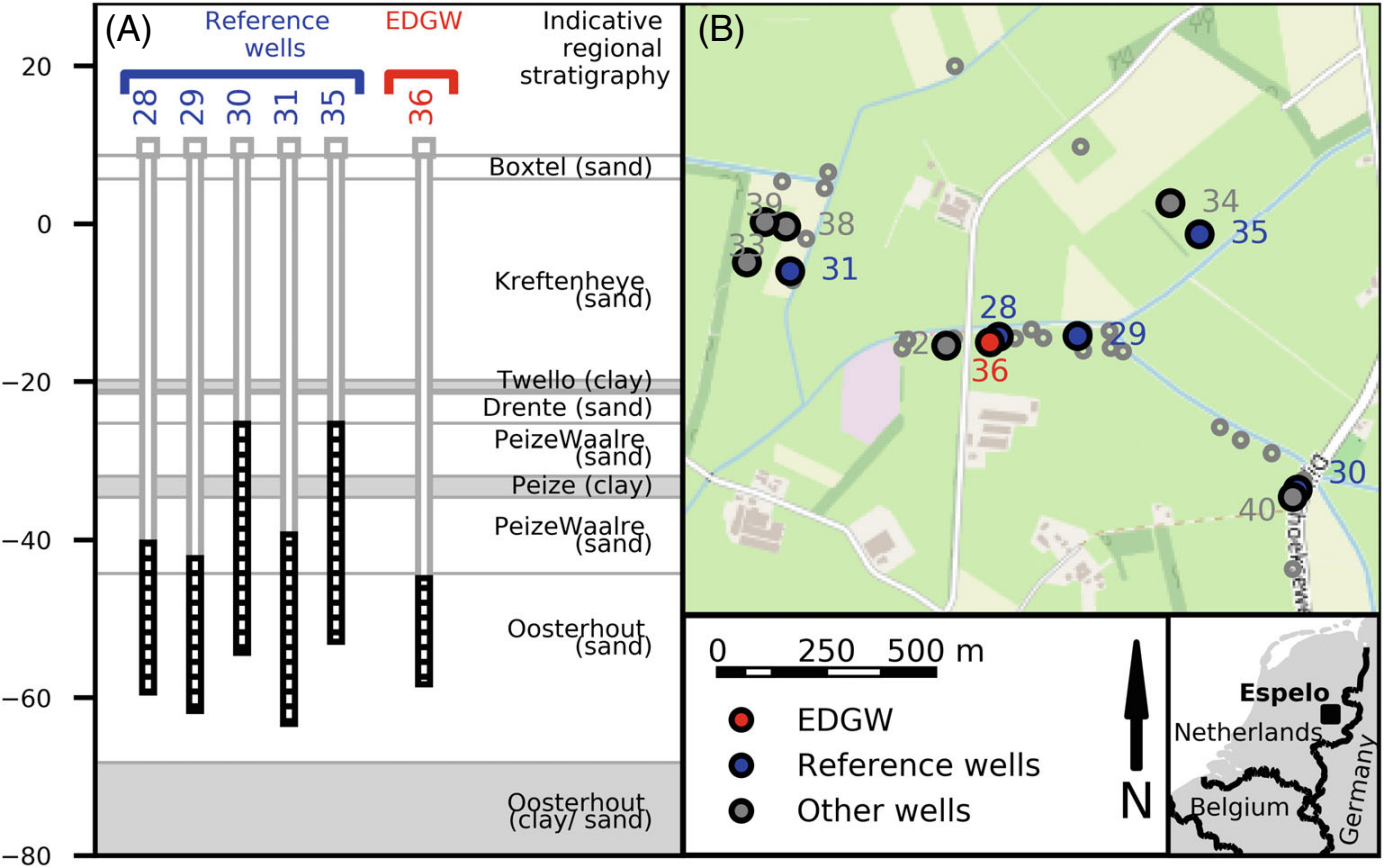
(A) Indicative regional stratigraphy with the dominant lithology of each formation at the EDGW location and the depth of the EDGW and surrounding wells. (B) Map with the location of the Espelo field site with the EDGW (red), the reference wells for monitoring clogging (blue) and other surrounding wells (gray). Reference wells are defined at the end of the methods section. Depth is displayed in m bgs.

We conducted a field trial to create an EDGW at a drinking water well field in Espelo, The Netherlands operated by Vitens (Figure 4). Conventional wells at this location have borehole diameters ranging from 600 to 900 mm and screen diameters from 200 to 315 mm. Many suffer from clogging by particle filtration at the borehole wall (Leunk 2012). Rehabilitation is required relatively frequent (typically every 3 year) and lifespan is relatively short (on average 15 years) compared with the range of 30 to 50 years for other Dutch well fields (van der Schans and Meerkerk 2020).

The target aquifer is located between 50 and 80 m bgs (below ground surface) (TNO 2020) and consists of unconsolidated medium to fine shallow marine sands of the Oosterhout formation. The bottom depth of the aquifer is variable and diffuse as the sands become finer. Groundwater in the aquifer is fresh (Cl 38 mg/L) and anoxic iron reducing (Fe^2+^ 3.5 mg/L).

### Construction of the EDGW in Espelo

First, a pilot borehole with a diameter of 600 mm was drilled using airlift reverse circulation drilling just into the top of the target aquifer at 52.5 m bgs, fitted with a casing and grouted in the annular space to prevent short circuit flow (Figure 2A). The pilot borehole was then drilled to a depth of 69.5 m bgs (Figure 2B). Next, the borehole diameter was expanded from 53.5 to 67 m bgs using the nozzle until it was extended 750 mm from the shaft center (Figure 2C). Finally, the well was completed by installation of a filter screen, gravel pack and seals, in a similar fashion as surrounding conventional wells, albeit with an extended diameter gravel pack. The borehole diameter was estimated in two independent ways (1) by recording the volume of formation material retained in the settling basin during drilling and (2) the amount of inserted gravel along with the elevation of the gravel pack during backfilling.

The experimental procedures for construction, development, operation, and rehabilitation are detailed in the Data S1.

### Development

The well was developed to stimulate removal of fine material from the borehole and surrounding formation. Next, a well test was performed by measuring the drawdown in the well while pumping 20 min with a volume flow of 60 m^3^/h. Comparison of heads in the well screen and a piezometer in the gravel pack confirmed that the well screen was not clogged initially.

### Operation

Production started in July 2016 with a volume flux of 60 m^3^/h. Pump scheduling was set up so that the submersible pump was switched on for 62% of the time (=utilization rate) and each on‐session had an average duration of 3.55 h (=operating period). The resulting velocity at the borehole wall was 16.1 m/d. The volume flux (*Q*) and heads (*H*) were continuously monitored with an automated pressure logger and flowmeter during operation.

### Rehabilitation with Acoustic Stimulation

To test if the larger diameter of the EDGW was limiting the impact of hydraulic rehabilitation, we tested if it was possible to rehabilitate the EDGW in June 2019, using a downhole low frequency (200 Hz) acoustic stimulation to overcome the barrier posed by the thick gravel pack. This unconventional experimental method relies on stimulating particle movement by the generation of resonance frequencies within the surrounding formation (Hartog and Westerhof 2010; van der Schans et al. 2014).

The acoustic stimulation was conducted twice, each time followed by chemical rehabilitation with acidified hydrogen peroxide. Surrounding wells often also receive two chemical and two mechanical rehabilitation steps. Drawdown and flow rate were measured and used to determine the specific volume flux after each treatment.

### Evaluation of Well Clogging

Well clogging was evaluated by comparing the specific volume flux (*Q*_*s*_) over time to the initial value after development of the well (*Q*_*s*,initial_) (van Beek et al. 2009):

(1) $$Q_{s}=\frac{Q}{s}$$

with *Q* being the volume flux and the drawdown (s) equal to the head difference immediately prior and a fixed duration after start of the pump (20 min for the field test in Espelo). However, the drawdown and hence specific volume flux are dependent on the dimensions of a well (radius and screen length), which makes *Q*_*s*_ not suited to compare the performance of the EDGW with conventional diameter wells. We therefore also defined the borehole entry resistance (*c*_*bh*_) which is independent of well dimensions and aquifer properties:

(2) $$c_{\mathrm{bh}}=\frac{K_{\mathrm{sk}}}{d_{\mathrm{sk}}}$$

Since it is practically impossible to measure the hydraulic conductivity (*K*_*sk*_) and thickness of the skin (*d*_*sk*_) directly during well operation (Houben 2015b), we instead deduced *c*_*bh*_ based on changes relative to the initial specific volume flux corrected for the borehole area (*A*_*b*_) (for derivation, see Data S2):

(3) $$c_{\mathrm{sk}}=A_{b}\left(\frac{1}{Q_{s}}-\frac{1}{Q_{s,\mathrm{new}}}\right)$$

*Q*_*s*,new_ was also used to estimate the hydraulic conductivity of the target aquifer (*K*_*aq*_) (see Data S3).

To assess the hydraulic performance of the EDGW, we selected reference wells that were drilled using a conventional diameter reverse rotary drilling (Figure 4) with aquifer and production characteristics similar to those of the EDGW. The *K*_*aq*_, median grain size (*d*_50_), utilization factor, average operating period, and velocity at borehole of reference wells varied less than 20% of the value found for the EDGW. The other (non‐reference) wells were located in coarser grained and more permeable formations and had a higher flow velocity (Table 1). Grain size and permeability are factors that have been shown to have a large influence on borehole clogging rates (e.g., de Zwart 2007).

**Table 1:** Hydraulic Performance of the EDGW, Reference Wells (with a *K*_*aq*_ < 13 m/d) and Other Wells Built in Espelo After 2010

Parameter	Symbol	Unit	EDGW (n = 1)	Reference Wells (n = 5)	Other wells (n = 6)
*Aquifer*					
Median grain size	*d*_50_	μm	196	196	292
Aquifer conductivity (apparent)	*K*_*aq*_	m/d	11.5	11.0	17.3
		m/s	1.3 × 10^−4^	1.3 × 10^−4^	2.0 × 10^−4^
*Well dimensions*					
Screen length	*L*_*sc*_	m	13.5	23.8	18.4
Borehole diameter	*D*_*bh*_	mm	1570	860	680
Borehole area	*A*_*bh*_	m^2^	72	63	38
*Operation*					
Velocity at borehole wall^1^	*v*_*b*_	m/d	16.1	15.3	31.0
		m/s	1.9 × 10^−4^	1.8 × 10^−4^	3.6 × 10^−4^
Pumping rate^1^	*Q*	m^3^/h	48	40	48
Utilization factor^1^	*U*	—	0.63	0.60	0.63
Average operating Period^1^	*t*_*op*_	h	3.6	4.1	4.9
Drawdown^1^	*S*	m	5.8	4.4	5.3
Specific flow rate^1^	*Q*_*s*_	m^2^/h	8.5	9.7	10.3
*Rehabilitation*					
Rehabilitation frequency^1^	*n*_*regs*_	1/yr	0.33	0.40	0.17

^1^ Average value during first 3 years of operation.

### Second Test with a Larger Borehole Expansion and Diameter

To test the ability to drill with an even larger borehole expansion, a second borehole was drilled in Espelo with the nozzle at 1230 mm from the drilling shaft. After successfully expanding the borehole diameter to at least 2460 mm (2 × 1230 mm) and jetting the top 8 m of the borehole to remove filter cake prior to backfilling, the borehole collapsed. It was backfilled after recovery of the drilling equipment.

## Results

### Borehole Geometry of the EDGW

**Figure 5:**
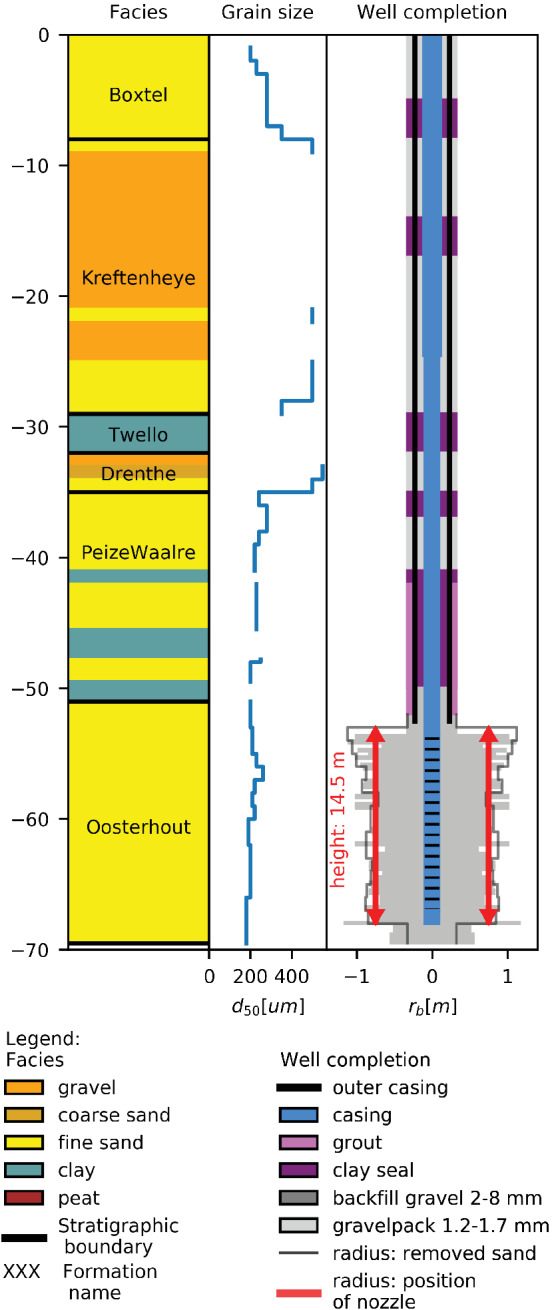
Drilling log and well completion of the EDGW at Espelo. The well radius is estimated based on both the volume of formation material removed from the borehole (gray line) and the volume of filter sand inserted in the well (gray polygon). Well radius scale is exaggerated relative to depth, displayed in m bgs.

Based on the amount of gravel added and regular monitoring of the gravel pack elevation during backfilling, the borehole created with the expansion nozzle had an average diameter of 1570 mm between 53.5 and 68 m bgs (Figure 5). Based on the volume of removed sand in the settling tanks, a larger average diameter of 1740 mm was calculated, which was likely an overestimation due to expansion of the formation sand due to decompression.

The estimated diameter based on backfilling varied with depth between 1210 and 2310 mm. Notably, the variation in material removed was smaller (1430–2130 mm). This could be due to small collapses during backfilling, variations in the settling slope of the gravel and/or horizontal drift of the plumb line used to measure the depth. There was some positive correlation (*r* ^2^ = 0.23) between the diameter and median grainsize of the formation (*d*_50_) and a trend for larger diameters toward the top of the expanded borehole. All‐in‐all, the estimated borehole diameter of 1570 (=785 mm radius) indicates that the borehole was eroded on average 35 mm beyond the position of the jetting nozzle at 750 mm from the borehole center.

### Hydraulic Performance During Well Construction and Development

During borehole expansion, the volume flux (*Q*) required to maintain the hydraulic overpressure (*s*) of 4 m was 3 m^3^/h during resting periods (*Q*_*s*_ = 0.75 m^2^/h). Water losses increased to 12 m^3^/h (*Q*_*s*_ = 4.0 m^2^/h) while attempting to cleanup the top meter of the borehole wall after drilling. This indicated that the jetting removed significant amounts of filter cake from the borehole wall. A larger initial *Q*_*s*_ would therefore be expected if the borehole would have been jetted over its entire height. The remaining part of the borehole was not jetted for fear that the water supply would be inadequate to prevent borehole collapse due to a broken alarm.

Initial pumping of the well during development led to an increase of *Q*_*s*_ from 0.75 to 9.9 m^2^/h and remained stable during all further development steps. The initial large increase of *Q*_*s*_ means that the borehole wall was significantly clogged when backfilling started, with a borehole entry resistance (*c*_*bh*_) of 3.7 d based on Equation 3. Since no drilling additives were used, clogging material likely consisted of natural fines mobilized from the sediment during the drilling. The hydraulic conductivity of the formation (*K*_*aq*_) based on well drawdown after development was 11.5 m/d (1.3 ·10^−4^ m/s) and in line with 8.46 to 12.01 m/d measured during Darcy experiments on disturbed soil samples (Speetjes 2016) and the *K*_*aq*_ found in surrounding wells with a similar median grain size (Table 1). Initial clogging, if present, was thus similar to the reference wells.

### Hydraulic Performance during the Operational Phase

Production rate per m well screen (*v*_*b*_ * *π* * *D*_*bh*_) was higher by a factor two in the EDGW compared with reference wells, in keeping with its nearly double borehole diameter. Due to the short length, the EDGW only had a 20% higher initial production rate (48 m^3^/h) compared with the reference wells (40 m^3^/h). The higher production rate also resulted in a larger drawdown since the well diameter itself has only limited influence on drawdown (e.g., Houben 2015a).

**Figure 6:**
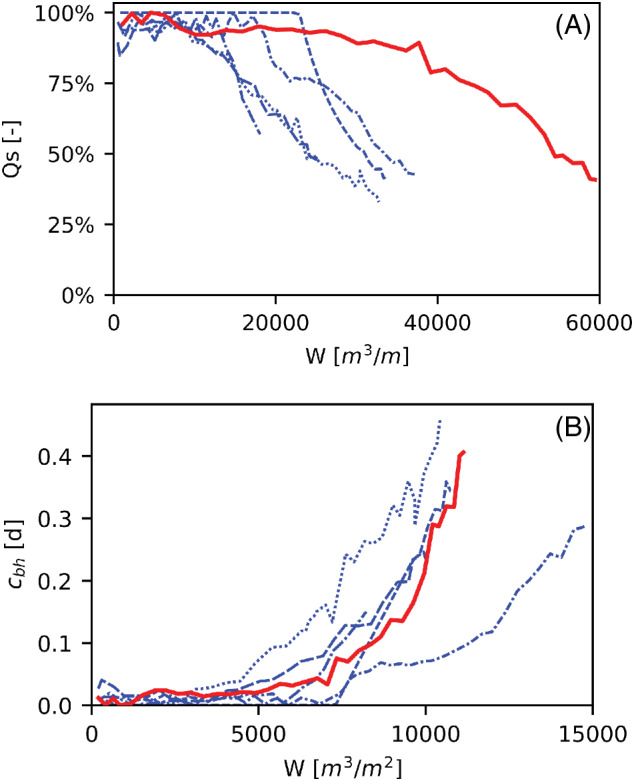
(A) Change in the specific volume flux as function of the abstracted volume per m well screen for the EDGW (red line) and reference wells (blue lines). (B.) Change in borehole entry resistance as function of the water influx over the borehole wall (= volume abstracted per m^2^ borehole surface area) for the same wells.

A total of 815,000 m^3^ water was abstracted between July 2016 and June 2019 when clogging of the borehole had reduced the *Q*_*s*_ to 42% of the original value and the well was regenerated. The abstracted volume per m well screen depth (*W* in m^3^/m) shows that the EDGW extracted only 60,000 m^3^/m before the specific volume flux was reduced to 50% of its initial value (Figure 6A, red line). The abstracted volume to this point was about two times larger than for the reference wells (Figure 6A, blue lines), in keeping with its two times larger borehole diameter.

The borehole resistance (*c*_*bh*_) initially remained stable with the volume abstracted per m^2^ borehole area (*w* in m^3^/m^2^) until it started to rise slowly after pumping 5000 m^3^/m^2^. The clogging rate accelerated after 9000 m^3^/m^2^ water influx. The clogging rate of the EDGW as a function of water influx fell within the range of the reference wells (Figure 6B). There was no increase of head difference between the casing and piezometer in the gravel pack or other indication of clogged well screens.

### Hydraulic Impact of Rehabilitation

**Figure 7:**
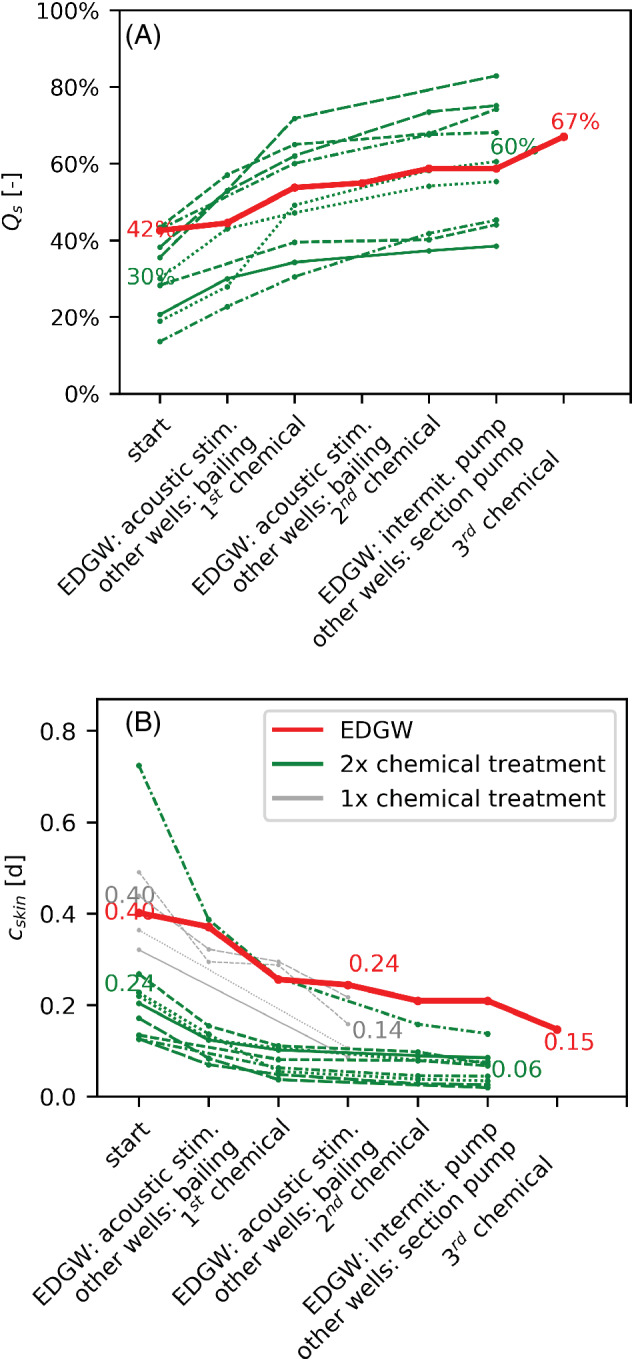
Impact of rehabilitation steps on the *Q*_*s*_ (A) and *c*_*bh*_ (B) for the EDGW (red line) and conventional wells that received either 2 chemical treatments (green lines) or 1 chemical treatment (gray lines). Note that the conventional wells are not the same reference wells as in Figure 6.

The rehabilitation of the EDGW resulted in a 25% improvement of the *Q*_*s*_ from 42% to 67% of *Q*_*s*,new_ (Figure 7A). This was slightly smaller than the 30% improvements of *Q*_*s*_ found for conventional wells at Espelo that also received two chemical treatments. However, the rehabilitation of the EDGW led to a much larger improvement (reduction) of the hydraulic resistance *c*_*bh*_ of 0.25 d (0.40–0.15, Figure 7B) compared with the 0.18 d (0.24–0.06) found at conventional wells. Note that *c*_*bh*_ is a more objective parameter to evaluate clogging of large diameter wells than *Q*_*s*_/*Q*_*s*,new_ since it is not influenced by the diameter of the borehole.

Water quality samples taken during rehabilitation demonstrate that acoustic stimulation caused an increase of suspended solids (TSS) from 6 to 289 mg/L while dissolved solids (TDS) only slightly increased from 522 to 527 mg/L. Chemical treatment resulted in a smaller increase of TSS (210 mg/L) but sharp increase of TDS to 1401 mg/L. Acoustic stimulation thus led to a greater release of particles from the well than chemical rehabilitation, especially when we consider that each acoustic stimulation cycle of 15 min was repeated eight times and chemical rehabilitation only once. The timing of the increased turbidity coincided with the travel time from the skin layer to the sampling point which suggests that clogging material is released from the borehole. However, it did not result in a strong reduction of the hydraulic resistance of the skin layer. Apparently the clogging was also caused by substances that were better removed by chemicals than mechanical rehabilitation, indicating that high turbidity during rehabilitation does not have to be an indication for effective rehabilitation.

## Discussion

### Potential Applications of Expanded Jetting and EDGW's

Results of the field study in Espelo showed that the jetting volume flux of 11 m^3^/h from the expandable nozzle resulted in an average radius of the borehole of 35 mm beyond the jetting arm radius of 750 mm. Although a smaller volume flux could reduce that separation distance when drilling in this aquifer, this would also increase the risk of physical contact between the nozzle and borehole. Oppositely, a larger jetting volume flux could result in removal of sediment further from the nozzle increasing the risk of unintended breakouts. The jetting volume flux required to prevent mechanical drag and breakouts will thus vary depending on the consistency and variability thereof over the entire expanded depth range. In order to maintain a constant distance between the nozzle and the borehole, this distance would need to be constantly monitored, for example, by means of a sensor mounted on the extension arm, and pressure (nozzle volume fluxes) adjusted accordingly.

### Borehole Stability

While a 1570 mm diameter borehole was used to construct the EDGW, a 2470 mm diameter borehole remained stable after drilling. The second borehole collapsed after 1 day, but only during a final attempt during which we removed a much larger section of the filter cake by jetting (8 m) compared with the first borehole (1 m) in order to minimize initial clogging of the borehole wall. Maintaining borehole stability requires a minimum pressure gradient over the borehole wall that is generally achieved by maintaining overpressure in the borehole compared with the surrounding formation in combination with the formation of a filter cake (Driscoll 1986). However, the minimally required pressure gradient can also be generated solely by maintaining sufficient overpressure in the borehole (Timmer 1998), as illustrated by scrape drilling which involves replacing the drilling fluid with drinking water followed by removing (underreaming) the entire skin layer before backfilling (Olsthoorn and Harlingen 1994). Perhaps the observed increase of water losses from 3 m^3^/h to approximately 25 to 30 m^3^/h in the second EDGW was not enough to maintain the minimal pressure gradient at the borehole wall required for stability. Note that the conditions for maintaining stability for the second borehole were aggravated by maintaining a lower overpressure (3 m) compared with the first borehole (4 m). Also, in the second borehole we replaced the drilling fluid with drinking water to prevent the reformation of a filter cake during final jetting. This replacement resulted in a lower fluid density in the borehole and thus a decrease of the overpressure. Additionally, decreasing borehole stability is associated with larger diameters, thus necessitating a higher minimum overpressure for larger boreholes (Timmer 1998; Papachimos 2010). This would seem especially the case for expanded boreholes due to the span required to support the downward vertical pressure at the roof of the expanded depth range. Overall, there is little insight into the critical boundary conditions that ensure stability during borehole expansion in unconsolidated formations.

### Timing of Rehabilitation

Unfortunately, due to the large diameter and hence borehole area, the large buildup of hydraulic resistance at the EDGW's borehole resulted in a much lower reduction of drawdown (and hence *Q*_*s*_) compared with wells with conventional diameter. The severe clogging was therefore noticed too late. Criteria to regenerate a well based on a reduction of *Q*_*s*_ compared with the original value recommended by, for example, Driscoll (1986) and Houben and Treskatis (2007) can thus not be directly applied to wells with larger diameters.

### Effectiveness of Mechanical Rehabilitation

Attempts to regenerate the EDGW illustrated that wells with larger diameters are harder to regenerate. We expected little effectiveness from regenerating the EDGW with conventional methods such as section‐wise pumping and bailing. The factor two thicker gravel pack would reduce the amount of energy reaching the borehole wall by at least a factor four as energy dissipated quadratically with distance, thus limiting the amount of water that moves into and out of the formation at clogged places that require stimulation (Driscoll 1986). Use of vibrations was thus aimed at stimulating the entire borehole wall, including clogged zones. The main difference with previous studies (Champion et al. 2004; Wong et al. 2004) is that we used frequencies in the acoustic (200 Hz) instead of ultrasonic range (10,000 Hz) to increase the penetration depth. Tests performed in a formation with similar diameter grainsize had demonstrated that penetration depth increases from several centimeters for ultrasonic stimulation (Bunnik 2004) to 10 m for acoustic stimulation (van der Schans et al. 2014), thus more than enough to reach the borehole wall with a downhole apparatus. However, we found that despite the removal of fines as indicated by the elevated suspended solids, acoustic stimulation had little impact on the EDGW's specific volume flux. This was a noticeable difference with bailing of the conventional wells which led to a substantial increase of the specific capacity (Figure 7B).

Compared with the EDGW, the other two times regenerated conventional wells were much less clogged before rehabilitation, thus making it hard to compare the rehabilitation efficiency. We therefore also evaluated the rehabilitation efficiency of wells that received only one chemical treatment and that had a c_bh_ similar to the EDGW between 0.3 and 0.5 d (average 0.4 d) (=gray lines in Figure 7B). After the first chemical treatment, more hydraulic resistance had been removed from the conventional wells (*C*_*bh*_ = 0.14) compared with the EDGW (*C*_*bh*_ = 0.24). The EDGW only reached these removal levels after three chemical treatments, suggesting that an EDGW would require three times more treatment steps compared with conventional wells. However, during each treatment the EDGW received 50% less chemicals per m^2^ borehole compared with the conventional wells. This occurred by accident, because the dose was only based on the length of the well screen without taking into account borehole diameter. Correcting for the 50% lower dose per m^2^ borehole implies that the EDGW would require only 1.5 times more treatment steps compared with conventional wells if chemicals were properly dosed.

Due to the severity of the clogging, we were not able to properly determine the additional costs and potential success rate of regenerating EDGW's if done timely. The difference in mechanical rehabilitation technique and dosing of chemicals compared with conventional wells makes it difficult to arrive at firm conclusions about the chances of successfully regenerating an EDGW compared with a conventional well. However, it is clear that more cleaning steps and chemicals are required, leading possibly (author's best guess) to a factor two higher costs.

### Screening of Economic Potential of the EDGW

Although the technical potential of the EDGW seems promising, its further development and application depends strongly on its economic potential. Based on indicative generic costs estimates (and assuming a 20 m long filter screen; see Data S4), we found that the construction costs of an EDGW (with diameter expanded from 600 to 1700 mm) and wells drilled with a large diameter (1700 mm) are higher than conventional wells (850 mm diameter) and backreamed wells (with diameter expanded from 425 to 850 mm). However, doubling the gravel pack diameter from 850 to 1700 mm leads to a doubling of the borehole area and hence a doubling of the production rate (assuming an equal entrance velocity at the borehole). Our example suggests that below approximately 80 m bgs, it could become economic to drill an EDGW when costs are normalized to borehole diameter. Larger expanded diameters would lead to even lower costs per cubic meter and hence an EDGW could already become economically attractive at lower depths.

We estimated that operational costs for an EDGW are comparable to a conventional well when normalized to the diameter. Energy costs due to drawdown (not to be confused with energy costs in case of a deep static groundwater table) generally comprise such a small fraction of operational costs (van der Schans et al. 2015), that the increased drawdown caused by higher volume fluxes has only limited financial impact. When assuming that the costs of regenerating an EDGW are two times larger than a conventional well, rehabilitation costs are similar to those for a conventional well when normalized by the borehole diameter.

Not included in our economic assessment are the additional risks associated with both borehole collapse and a shorter lifetime due to challenges regenerating the larger borehole. Better means to estimate and manage these risks is thus required to make an informed decision of when to apply the EDGW‐technique.

## Conclusions

In this study, we developed and field‐tested a novel drilling technique that employs an extendable jetting nozzle to enlarge the borehole diameter at target depth range in unconsolidated formations in a controlled manner. During field testing, the borehole diameter was expanded 2.6‐fold from 600 to 1570 mm at a depth of 53.5 to 68 below ground surface. The borehole diameter was only 70 mm larger than the 1500 mm expected based on the position of the jetting nozzle. This means that the extendable perpendicular jetting with the applied pressures allowed control over the borehole diameter for the Espelo conditions. Subsequently, a well screen was placed and the remaining borehole was gravel packed to create the EDGW, after which it was taken in routine operation for drinking water production. Initial abstraction confirmed that the larger diameter allowed a higher discharge rate compared with conventional wells while maintaining a similar flow velocity at the borehole. In keeping with the larger borehole area, we were able to extract double the amount of water compared with conventional reference wells before clogging caused a noticeable increase in drawdown and a similar amount after normalization of abstracted volumes by the borehole area. Rehabilitation of the EDGW was less successful, partly because the large diameter limited the additional drawdown and thus obscured that the borehole was getting heavily clogged. Large‐diameter wells thus require close monitoring. We were not able to draw definite conclusions as to whether the EDGW is suitable for rehabilitation.

The potential benefits of the EDGW are largest in deep formations, especially when construction costs makeup a large portion of the total cost of ownership due to limited lifetime of wells. The technique may thus also contribute to water and energy sustainability through the cost‐effective construction of ASR and ATES wells in deeper aquifer. Further understanding of the potential to remove the filter cake during construction and rehabilitation is required to assess its applicability under a wider range of aquifer characteristics.

## Conflict of Interest

The co‐author N. Robat is an employee of the drilling company Haitjema B.V. that built and owns the extendable drilling bit developed in this study.

## Supporting information


**Data S1.** Detailed description of field procedures during the EDGW field trial.
**Data S2.** Procedure to estimate the entry resistance of the borehole wall based on specific volume flux.
**Data S3.** Procedure used to estimate the hydraulic conductivity of a semi‐confined aquifer using steady state pumping tests in partially penetrating wells.
**Data S4.** Cost comparison between the EDGW and other well types.
