# Supplementary material for: Field Testing of a Novel Drilling Technique to Expand Well Diameters at Depth in Unconsolidated Formations
Source: Ground Water. 2022 May 6;60(6):808–19. doi: 10.1111/gwat.13203 (PMC9790686; doi:10.1111/gwat.13203)
Supplement: Supplementary file 1 — Data S1. Detailed description of field procedures during the EDGW field trial. Data S2. Procedure to estimate the entry resistance of the borehole wall based on specific volume flux. Data S3. Procedure used to estimate the hydraulic conductivity of a semi‐confined aquifer using steady state pumping tests in partially penetrating wells. Data S4. Cost comparison between the EDGW and other well types. [file GWAT-60-808-s001.pdf]

Supporting information

Field Testing of a Novel Drilling Technique to Expand Well Diameters at Depth in Unconsolidated Formations

van der Schans, M.L.[1,2], M. Bloemendal[1,2], N. Robat[3], A. Oosterhof[4], P.J. Stuyfzand[5] , N. Hartog[2,6]

1. Faculty of Geoscience & Engineering, Delft University of Technology, The Netherlands.
2. KWR Water Research Institute, Nieuwegein, The Netherlands.
3. Haitjema B.V., Dedemsvaart, The Netherlands.
4. Vitens N.V., Zwolle, The Netherlands.
5. Hydroconsult+, Zandvoort, The Netherlands.
6. Department of Earth Sciences, Utrecht University, Utrecht, The Netherlands.

S1: Detailed description of field procedures during the EDGW field trial

S1.1 Construction of the EDGW in Espelo

The EDGW at Espelo was constructed through the following steps:

1. A settling basin was placed on a plateau, to enhance borehole pressure and thus stability, so that the water table was 2 m above ground surface or 4 m above the static groundwater head of 2 m bgs.
2. Next, a pilot borehole with a diameter of 600 mm was drilled using air-lift reverse circulation drilling just into the top of the target aquifer at 52.5 m bgs.
3. An outer PVC casing of 500/ 468.1 mm diameter (supplied by Boode) was installed in the borehole to a depth of 51 m bgs to. The annulus was grouted to prevent short circuit flow to the overlying aquifer around the casing, stabilize the formation above the expanded borehole and keep the casing in place during jetting.
4. The pilot borehole was then drilled to a depth of 69.5 m bgs.
5. The borehole was expanded from 53.5 to 67 m bgs. The top 2 m of the aquifer were not expanded to prevent accidental erosion of the confining clay layer. The depth interval from 68 to 69.5 m was used to install a clay plug and a sand trap.
6. The expansion was done in vertical downward steps of one meter that each took approximately 30 minutes to complete until reaching the 750 mm distance between nozzle and shaft center. The force needed to rotate the agitation arm was closely monitored and did not indicate collapse or contact with the formation.
7. The drilling and expansion were conducted without drilling additives (no bentonite, etc.). Once the desired depth was reached, the drilling fluid in the borehole and settling basin were replaced by clean groundwater from nearby wells to limit initial clogging of the borehole as is common practice for Dutch ATES wells (SIKB 2014).
8. After expanding the entire borehole, we started jetting the borehole wall from top to bottom to remove remaining clogging material (skin). We stopped after cleaning the top 1 m due to the strong increase in water loss from 3 m^3 to 12 m^3/h. It was feared that the water supply would not be adequate to prevent borehole collapse due to a broken water supply alarm system.
9. Well completion was done in a similar fashion as surrounding conventional wells and according to the prevailing Code of Practice for the Dutch Water sector (Makkink et al. 2011)and Dutch drilling standard (SIKB 2018). In short, a PVC well screen (manufactured by Boode) was installed over the depth range of the expanded diameter with a diameter of 200 mm and 1 mm slots. The inner casing had a similar diameter. The gravel pack was installed with a tremie (diameter 101 mm) lowered into the annulus. The median grain size was 1.4 mm, range 1.2 to 1.7 mm, which is factor 7 larger than the 196 µm median grain size (d50) in the surrounding

formation and similar to surrounding wells. Next, the well was pumped to check if there was no delayed settling of the gravel. Once this was confirmed, the annulus between the inner and outer casing was filled with backfilling gravel (grainsize 2-8 mm) and clay pallets (Mikolit 00 supplied by Terratech) as sealing material at the depth interval of confining layers.

Monitoring of the construction process encompassed recording a drilling log using the Dutch standard (NEN 2019). We also registered the water level in the settling basin and volume flux of work water supply. The size of the borehole was checked every meter by comparing the diameter according to the position (distance) of the nozzle from the drilling shaft with the volume of formation material retained in the settling basin. The amount of gravel was regularly determined along with measuring the elevation of the gravel pack during backfilling. This yielded two independent estimates of the borehole diameter and its vertical variation.

S1.2 Development and initial testing

The well was developed to stimulate removal of fine material from the borehole and surrounding formation by a stepwise increase of the flow rate for several days until it reached 90 m^3/h. Next, the well was developed by intermittent abstraction (for half a day at 60 m^2/h with 10 minute intervals followed by 5 minute restperiod) and bailing (20x with 2 minute intervals at 1 bar and brief abstraction with 60 m^2/h every 20 minutes). Then, 3 m^3 H_2O_2 (10%) and HCl (1%) was added. These chemicals were the same as applied in regenerations of neighboring wells. This was followed by adding 7 m^3 native ground water (to displace the chemicals to the clogging material at the borehole wall) and intermittently adding and pumping 3 m^3 water to facilitate proper mixing and uptake of the chemicals. After waiting 16 hours, the well was pumped clean through intermittent pumping with its nominal flow rate of 60 m^3/h for 18 hours (27 minutes on; 14 minutes off).

Before and after each development step, a well test was performed by measuring the drawdown in the well whilst pumping 20 minutes with a volume flow of 60 m^3/h. Comparison of heads in the well screen and a piezometer in the gravel pack confirmed that the well screen was not clogged initially. We also performed a pumping tests with piezometers at 11 and 30 m distance. However, upon analysis of the data we found that the initial heads were not static due to a lack of resting time and disruption by on- and off-switching of pumps in neighboring wells. This prevented a proper assessment of the potential specific capacity.

S1.3 Operation

Production started in July 2016 with a volume flux of 60 m^3/h. Pump scheduling was set up so that the submersible pump was switched on for 62% of the time (=Utilization rate) and each on-session had an average duration of 3.55 hours (=Operating period). The resulting velocity at the borehole wall (16.1 m/d), Utilization rates and Operating periods were similar to the neighboring wells. In 2018 and 2019, the flow rate was gradually reduced to 40 m^3/h in an unsuccessful attempt to reduce further clogging. The volume flux (Q) and heads (H) were continuously monitored with an automated pressure logger and flowmeter during operation.

S1.4 Regeneration

On the first day, acoustic stimulation was applied for 8 cycles that each consisted of 3 phases of 45 (3x15) minutes. During each cycle the vibrating element was first switched on and continuously moved up and down the entire well screen while pumping to remove displaced particles. Next, the well was clean pumped (40 m^3/h) without vibration. This was finally followed by a resting period without pumping. At the end of the first day, 1.35 m^3 10%H2O2+1%HCl (=0.1 m3/m well screen) was applied followed by addition of 10 m^3 native groundwater to displace the chemicals to the borehole and 4 hours of bailing (2 bar). On day 2, the well was clean pumped followed again by 8 cycles of acoustic stimulation on day 5. On day 6, 2.7 m^3 10%H2O2+1%HCl was applied followed again by addition of 10 m^3 water, bailing and 10 hours intermittent clean pumping the next day (10 minutes on; 5 minutes rest). On day 7, a third dose of 1.35 m^3 chemicals was applied but with a higher dose (20%H2O2+1%HCl). This was also again followed by addition of native groundwater, bailing and 12 hours intermittent pumping.

Continuous measurements of flow rate and drawdown were conducted throughout the regeneration. Physical and chemical parameters were measured several times before and during acoustic stimulation and clean pumping the well from chemical regeneration to determine the reactants in the skin and surrounding aquifer.

S1.5 Second test with a larger borehole expansion and diameter

To test the ability to drill with an even larger borehole expansion and its resulting borehole stability, a second borehole was drilled at approximately 100 m distance from the first EDGW over the same depth range. The pivoting angle and length of the expansion arm were increased so that the nozzle could expand to 1230 mm from the drilling shaft instead of 750 mm. The settling basin was placed on the ground, thus reducing overpressure to 3 m instead of 4 m, but still above the 2 m minimum

recommended by Dutch codes of practice (Makkink et al. 2011, BodemenergieNL 2013). During expansion, the settling basin was supplied entirely with clean groundwater instead of recycled drilling fluid. After successfully expanding the borehole diameter to at least 2460 mm (2x 1230 mm), we began a final round to jet the expanded borehole wall from top to bottom and remove filter cake prior to backfilling. After jetting the top 8 m, the water uptake was approx. 40 m^3/h. At this point, water level fluctuations of several decimeters started to occur in the settling basin (observed by the drilling crew) which indicated pressure instability in the borehole. The borehole collapsed shortly after and a second EDGW could not be completed.

S2: Procedure to estimate the entry resistance of the borehole wall based on specific volume flux

Head losses near the borehole wall of a well (s_{sk}) due to the formation of a filter cake and decrease of hydraulic conductivity in the damage zone around the borehole (here together defined as skin layer) are a function of the volume flux (Q), screened depth interval (D), borehole diameter (r_b), hydraulic conductivity of the skin layer (K_{sk}) and thickness of the skin layer (d_{sk}). Assuming an even distribution of the filter cake and laminar flow therein, these losses can be approached by the Theim-equation as adapted by Houben (2015):

$$s_{sk} = \frac{Q}{2\pi K_{sk} D} ln\left(\frac{r_b + d_{sk}}{r_b}\right) \tag{S2.1}$$

Since the most severely clogged zone is generally thin compared to the borehole radius (Timmer et al. 2003, de Zwart 2007, Houben et al. 2016, Weidner et al. 2016), we may assume that:

$$ln\left(\frac{r_b + d_{sk}}{r_b}\right) \cong \frac{d_{sk}}{r_b} \tag{S2.2}$$

The error margin of this relation is below 10% so long as d_{sk} is smaller than r_{bh}/4. Next, we define the borehole entry resistance (c_{bh}) as:

$$c_{sk} = \frac{d_{sk}}{K_{sk}} \tag{S2.3}$$

and the borehole wall area (A_b) as:

$$A_b = 2\pi r_b D \tag{S2.4}$$

Combining and reorganizing equations S2.1 through S2.4 yields the following relation:

$$s_{sk} = \frac{Q c_{sk}}{A_b} \tag{S2.5}$$

We further assume that any increase of total well losses (s_t) compared to losses immediately after initial developing of the well (s_{new}) is due to skin losses caused by particle clogging at or around the borehole wall:

$$\frac{s_{sk}}{Q} = \frac{s_t}{Q} - \frac{s_{new}}{Q} \tag{S2.6}$$

Combining and reorganizing equations S2.5 and S2.6 and replacement of Q/s by the specific volume flux (Q_s) gives a simple equation to approximate the borehole entry resistance based on changes to the specific volume flux compared to its initial value ($Q_{s,new}$):

$$c_{sk} = A_b\left(\frac{1}{Q_s} - \frac{1}{Q_{s,new}}\right) \tag{S2.7}$$

Note that this approach results in an “apparent” c_{sk}, as it does not take into account head loss due to any initial clogging of the well already present immediately after well development. In the case that the formation is developed spontaneously during operation (van Beek et al. 2009), c_{sk} becomes negative over time.

S3: Procedure used to estimate the hydraulic conductivity of a semi-confined aquifer using steady state pumping tests in partially penetrating wells

To estimate the hydraulic conductivity (K_{aq}) of a semi-confined target aquifer, using steady state pumping test data from a partially penetrated groundwater well, we used a similar approach as Houben (2015). However, instead of relying on the Thiem equation, we combined the de Glee equation (Kruseman and De Ridder 2000) with the equation of Barker and Herbert (1992) to compute aquifer losses due to partial penetration as follows:

$$K_{aq} = \frac{Q_S}{2\pi B_{aq}}\left[K_0\left(\frac{r_b}{\lambda}\right) + \frac{(1-p_p)}{p_p}\cdot ln\left[\frac{p_p(1-p_p)}{(2-\varepsilon^2)}\frac{B_{aq}}{r_b}\sqrt{\frac{K_h}{K_v}}\right]\right] \quad \text{(S3.1)}$$

In this equation, K_0 stands for a zero-order Bessel function and the partial penetration factor (p_p) is the quotient of the screen length (L_{sc}) and B_{aq}. The eccentricity ε is computed as:

$$\varepsilon = \frac{2z_c}{B_{aq}(1-p_P)} \quad \text{(S3.2)}$$

With z_c the distance from the top of the well screen to the overlying confining layer and B_{aq} the thickness of the aquifer. The influence distance (λ) depends on the vertical conductivity (K_{conf}) and thickness (B_{conf}) of the overlying confining layer:

$$\lambda = \sqrt{K_{aq}B_{aq}\frac{B_{conf}}{K_{conf}}} \quad \text{(S3.3)}$$

Equation S3.1 needs to be solved iteratively since λ depends on K_{aq}. Note that this method assumes that the measurements are conducted in a well that is not clogged and has very low losses in the gravel pack and well screen.

In Espelo, other well losses are assumed negligible based on initial computations using the methods presented by (Houben 2015) and comparison of heads in the pumped casing and a piezometer in the gravel pack. But since we could not assess whether the EDGW and other wells in Espelo were completely developed and thus not influenced by initial skin losses, it is perhaps more accurate view K_{aq} as the “apparent” hydraulic conductivity. Also, since the drawdown was measured after 20 minutes of pumping, we neglected a small amount of drawdown that would occurred until reaching steady state. The B_{conf}/ K_{conf} at Espelo was estimated at 1000 d based on drilling logs and the regional hydrogeological model, using the Oosterhout clays as hydrogeological base (TNO 2020).

S4: Cost comparison between the EDGW and other well types

Table S4.1 *Indicative comparison of unit costs for construction and operation of a conventional well with 850 mm diameter, a well with a large diameter drilled over the entire depth (1700 mm), a borehole expanded with an underreamer (from 425 to 850 mm) and an EDGW (600 mm pilot borehole and 1700 mm expanded borehole) based on figures provided by Vitens and Haitjema. The remarks in the last 3 columns explain why costs are different from a conventional well.*

Cost aspect	Well type	Conventional well	Remark *	Large dia- meter well	Remark **	Under- reamer	Remark **	EDGW	Remark **	
		diameter: 0.85 m		diameter: 1.7 m		diameter: 0.425 -> 0.85 m		diameter: 0.6 -> 1.7 m		
Construction										
Generic construction costs	k€/well	100	Transport and build-up of drilling material, well development,	110	Larger capacity submersible pump, additional working	105	Additional worki	130	Larger capacity submer	A
Unit costs in overburden	k€/m	0	Drilling, permant casing, sealing, disposal of soil	1	Extra drilling, sealing material and soil disposal	0.2	Less drilling, sealing material and soil disposal	0.5	Purchase (200 €/m), install outer casing, cheaper drilling	B
Unit costs in target aquifer	k€/m	1	Drilling, filter screen, gravel, section pumping (other	2	Extra drilling, gravel and development	2	Extra drilling, gravel and development	2.5	Reaming borehole, extra gravel and development	C
Operation & maintenance										
Generic operation costs	k€/well/y	10	Operational staff, pump replacement,	12.5	Heavier pump more expensive to replace	10		13	Heavier pump more expensive to replace	D
Unit costs Electricity	k€/kwh	0.0008		0.0008		0.0008		0.0008		E
Unit costs rehabilitation	k€/event	15	mechanical and twice chemical treatment	30	twice more treatment steps and chemicals	15		30	twice more treatment steps and chemicals	F
(*) Explanation of costs (**) Explanation why costs are different from a conventional well										

Table S4.2 *Indicative computation of the construction costs and operational costs of an EDGW and other well types with a filter length of 20 m and based on generic costs and dimensions outlined in Table S4.1. The letters A thru F also refer to Table S3.1.*

Cost aspect \ Well type		Conventional well	Large diameter well	Under-reamer	EDGW		Remark on computation
Scenario's: construction costs							
Diameter gravelpack	m	0.85	1.7	0.85	1.7	G	
Length filterscreen	m	20				H	
End depth	m	Construction costs (k€)				J	A+(J-I)*B+I*C
	20	120	150	145	180		
	100	144	230	161	220		
	200	174	330	181	270		
	300	204	430	201	320		
	400	234	530	221	370		
	500	264	630	241	420		
		Construction costs, normalized by diameter (€/m)				K	J/G
	20	141.18	88.24	170.59	105.88		
	100	169.41	135.29	189.41	129.41		
	200	204.71	194.12	212.94	158.82		
	300	240.00	252.94	236.47	188.24		
	400	275.29	311.76	260.00	217.65		
	500	310.59	370.59	283.53	247.06		

Well type / Cost aspect	Conventional well	Large dia-meter well	Under-reamer	EDGW		Remark on computation
Scenario's: operational costs						
hydraulic condu m/d	Flow velocity at borehole (m/d)				L	
10	31.0				M	86400*sqrt(L/86400)/30
25	49.0					
50	69.3					
75	84.9					
100	98.0					
	Volume Flux (m3/d)					
10	1,654	3,308	1,654	3,308	N	2*3.14*G*H*M
25	2,615	5,230	2,615	5,230		
50	3,698	7,397	3,698	7,397		
75	4,529	9,059	4,529	9,059		
100	5,230	10,460	5,230	10,460		
	Hydraulic resistance of confining layer (days) = thickness / hydraulic conductivity					
	1,000				O	
	Drawdown					
10	10	19	10	19	P	L*BesselK$_0$(0.5*G/O)/(2*3.14*L*H)
25	7	12	7	12		De Glee (1930)
50	5	8	5	8		
75	4	7	4	7		
100	3	6	3	6		
	Utilization factor (%)					
	50%				Q	
	Annular production (m3/y)					
10	301,840	603,680	301,840	603,680	R	365*N
25	477,251	954,502	477,251	954,502		
50	674,935	1,349,870	674,935	1,349,870		
75	826,623	1,653,247	826,623	1,653,247		
100	954,502	1,909,005	954,502	1,909,005		
	energy efficiency of pump (%)					
	70%				S	
	Energy use (kWh)					
10	1,243	4,534	1,243	4,534	T	P*R/Q*9.81/3600
25	1,243	4,534	1,243	4,534		
50	1,243	4,534	1,243	4,534		
75	1,243	4,534	1,243	4,534		
100	1,243	4,534	1,243	4,534		
	Operational Costs (kE/y)					
10	11	16	11	16		
25	11	16	11	16		
50	11	16	11	16		
75	11	16	11	16		
100	11	16	11	16		
	Operational cost, normalized by borehole diameter (k€/y/m)					
10	12.93	9.49	12.93	9.49		
25	12.93	9.49	12.93	9.49		
50	12.93	9.49	12.93	9.49		
75	12.93	9.49	12.93	9.49		
100	12.93	9.49	12.93	9.49		

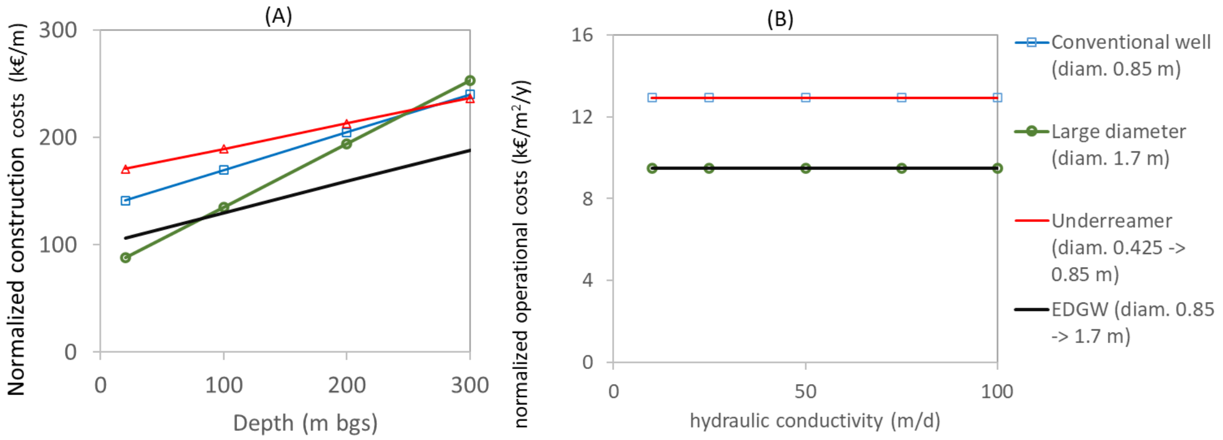

Figure S4.1. Indicative comparison of the construction costs (A) and operations costs (B) of a conventional well, large diameter well and an EDGW. Costs are normalized by the gravel pack diameter for a well with a filter screen of 20 m long as outlined in Table S4.1 and S4.2.

References

Barker, J. and R. Herbert. 1992. A Simple Theory For Estimating Well Losses: With Application To Test Wells In Bangladesh. Applied Hydrogeology 0: 20-31. https://doi.org/10.1007/PL00010959

BodemenergieNL. 2013. https://www.wikibodemenergie.nl/inhoudsopgave/open_bodemenergiesystemen/realisatie-open-bodemenergiesystemen/beheersing_boorproces/toepassing_boorspoeling/, accessed on: 15-10-2020

de Glee, G.J. 1930. Over grondwaterstroomingen bij waterontrekking door middel van putten [On groundwater flow near abstractions with wells]. Technische Hoogeschool Delft, Delft. https://edepot.wur.nl/413037

de Zwart, A.H. 2007. Investigation of clogging processes in unconsolidated aquifers near water supply wells. 1st edition. Ponaen & Looyen BV, Delft. http://resolver.tudelft.nl/uuid:ea1dc826-eb40-4eef-b03a-b59c5cd2047a

Houben, G.J. 2015. Review: Hydraulics of water wells—head losses of individual components. Hydrogeology Journal 23: 1659–1675. https://doi.org/10.1007/s10040-015-1313-7

Houben, G.J., M. Halisch, S. Kaufhold, C. Weidner, J. Sander and M. Reich. 2016. Analysis of Wellbore Skin Samples—Typology, Composition, and Hydraulic Properties. Groundwater 54, no.5: 634-645. https://doi.org/10.1111/gwat.12403

Kruseman, G.P. and N.A. De Ridder. 2000. Analysis and evaluation of pumping test data. 11, second edition. International institute for land reclamation and improvement The Netherlands. https://www.hydrology.nl/images/docs/dutch/key/Kruseman_and_De_Ridder_2000.pdf

Makkink, H.J., M.L.M. Balemans and I. Leunk. 2011. Kennisdocument Putten(velden), Ontwerp, aanleg en exploitatie van pomp- en waarnemingsputten [Knowledge document well(fields), design, construction and operation of production and observation wells]. KWR 2011.014, KWR, Nieuwegein. https://library.kwrwater.nl/publication/48690665/

NEN. 2019. Geotechnisch onderzoek en beproeving - Identificatie en classificatie van grond - Deel 1: Identificatie en beschrijving (incl. Nederlandse bijlage:2019)[Geotechnical research and testing - identification and classification of soils - part 1: identicication and description.].

SIKB. 2014. Protocol 11001: Ontwerp, realisatie en beheer van het ondergrondse deel van bodemenergiesystemen[Design, construction and operation of the subsurface part of aquifer thermal energy system]. Stichting Infrastructuur Kwaliteitsborging Bodembeheer, Gouda. https://www.sikb.nl/bodembeheer/richtlijnen/brl-11000

SIKB. 2018. Protocol 2101: Mechanisch Boren[Protocol 2101: Mechanical drilling]. Stichting Infrastructuur Kwaliteitsborging Bodembeheer, Gouda. https://www.sikb.nl/richtlijnen/brl-2100

Timmer, H., J.D. Verdel and A.G. Jongmans. 2003. Well clogging by particles in Dutch well fields. Journal-American Water Works Association 95, no.8: 112-118. https://doi.org/10.1002/j.1551-8833.2003.tb10434.x

TNO. 2020. https://www.dinoloket.nl/en/subsurface-models, accessed on: January 2020

van Beek, C.G.E.M., R.J.M. Breedveld, M. Juhász-Holterman, A. Oosterhof and P.J. Stuyfzand. 2009. Cause and prevention of well bore clogging by particles. Hydrogeology Journal 17, no.8: 1877. https://doi.org/10.1007/s10040-009-0537-9

Weidner, C., G. Houben, M. Halisch, S. Kaufhold, J. Sander, M. Reich and C. Menz. 2016. Wellbore Skin in Mine Dewatering and Drinking Water Supply: Field Observation, Mineralogy and Hydraulic Effect. In: Drebenstedt, C., Paul, Michael (Ed.), Annual Meeting of the International-Mine-Water-Association (IMWA), Freiberg/Germany: 478-485. http://www.mwen.info/docs/imwa_2016/IMWA2016_Weidner_195.pdf
